# A systematic review and meta-analysis of the effects of mind-body exercise on depressed and anxious individuals

**DOI:** 10.7717/peerj.20570

**Published:** 2026-01-06

**Authors:** Zheng Ye, Zhihui Xu, Xing Wang

**Affiliations:** 1 College of Sports Science, Shenyang Normal University, Shenyang City, Liaoning Province, China; 2 Faculty of Physical Education, Tomsk State University, Tomsk City, Tomsk Oblast, Russia; 3 School of Physical Education, Shanghai University of Sport, Shanghai, Shanghai, China

**Keywords:** Depression, Anxiety, Mind body exercise, Tai Chi, Yoga, Qigong

## Abstract

**Background:**

This systematic review evaluates the effectiveness of mind-body exercise in alleviating symptoms of depression and anxiety. It further compares the effects of different types of mind-body exercise and examines whether intervention cycle, session length, or frequency exhibit dose-response relationships.

**Methods:**

This study adhered to Preferred Reporting Items for Systematic Reviews and Meta-Analyses (PRISMA) guidelines and systematically searched seven prominent databases—Web of Science, PubMed, Cochrane Library, Embase, CNKI, WANFANG DATA, and the VIP database—from their inception through July 6, 2024. Only randomized controlled trials (RCTs) examining the impact of mind-body exercise interventions on depression and anxiety were included in the analysis. All experimental groups engaged only in mind-body exercise, and control groups received no intervention. Meta-analysis, subgroup analysis, sensitivity analysis, and assessment of publication bias were performed using Stata 17.0 software. Evidence quality was assessed using the GRADE tool.

**Results:**

A total of 15 studies that encompassed 1,351 participants were included in this review. The meta-analysis demonstrated that mind-body exercise significantly alleviated symptoms of depression (Hedges’ *g* = −0.86, 95% CI [−1.24 to −0.48], *P* < 0.001) and anxiety (Hedges’ *g* = −0.38, 95% CI [−0.53 to −0.23], *P* < 0.001). Five subgroup variables were examined in this study: exercise type, session duration (minutes), intervention period (weeks), frequency (sessions per week), and baseline depression severity. In the analysis of depression outcomes, exercise type, session duration, frequency, and baseline depression severity were identified as significant moderators. The most effective intervention characteristics for reducing depressive symptoms were: Qigong as the exercise type, sessions lasting 31–60 min, a frequency of three sessions per week, an intervention period of 9–12 weeks, and high baseline levels of depression. In contrast, none of these variables were found to be significant moderators in the analysis of anxiety outcomes. However, the most effective intervention characteristics for alleviating anxiety symptoms were tai chi as the exercise type, sessions lasting 31–60 min, a frequency of four or more sessions per week, an intervention period of 8 weeks or less, and normal baseline anxiety levels.

**Discussion:**

There was robust evidence that mind-body exercise significantly reduces symptoms of depression and anxiety.

**Other:**

This study adhered to PRISMA guidelines to ensure rigorous transparency and methodological accuracy. Furthermore, it was formally registered on the PROSPERO international systematic review platform (https://www.crd.york.ac.uk/prospero/) under registration number CRD42024613769.

## Introduction

Depression and anxiety are prevalent mental health disorders (Brinsley et al., 2022) that rank among the foremost contributors to disability on a global scale (Yang et al., 2021; Xu, 2024). The clinical presentations of depression and anxiety are multifaceted (Cosci & Fava, 2021), and these disorders are associated with cognitive impairments, including diminished attention, memory deficits, and reduced information processing speed (Freedman et al., 2024). These mental health conditions may culminate in severe outcomes, including self-harm and suicide (Kalin, 2021). The prevalence of depression and anxiety has been increasing steadily on an annual basis, and according to the World Health Organization’s Global Burden of Disease assessment, depression is projected to become the leading cause of disease burden worldwide by 2030 (Xiong et al., 2022; Zhang et al., 2024). From 1990 to 2021, the prevalence of anxiety has shown an upward trend, and it is estimated that by 2050, individuals aged 15–19 will experience the highest prevalence of anxiety disorders (Chen, Du & Zhu, 2025).

Contemporary research underscores that exercise serves as an effective intervention for mitigating symptoms of depression and anxiety (Wang, 2022). In comparison to pharmacological and psychological treatments, exercise offers a cost-effective, accessible, and non-toxic alternative that simultaneously enhances mood (Liu et al., 2024). Notably, mind-body exercises exhibit comparable effectiveness to pharmacological treatments in alleviating symptoms (Rådmark et al., 2020). Mind-body exercises integrate physical movement with mental focus and emphasize body–mind unity through slow movements, deep breathing, and mindfulness or meditation to promote relaxation and balance (Li et al., 2024; Zhang, Cao & Yan, 2025). Mind-body exercises regulate attention and enhance bodily awareness by directing focus to breathing and proprioceptive sensations (Goldbeck et al., 2021). Qigong, tai chi, and yoga are representative forms of mind-body exercise. These practices typically incorporate postures, breathing techniques, and meditation, and are among the most common types of mind-body exercises (Gauthier et al., 2022). Unlike aerobic and resistance exercises, mind-body exercises integrate muscular activity with a deliberate internal focus to cultivate self-meditative states. This method encompasses both physical activity and additional components such as breath control and psychological relaxation (Zhang, Liu & Gao, 2022). This approach has been shown to effectively attenuate pro-inflammatory cytokines, augment levels of brain-derived neurotrophic factor (BDNF) (Sun et al., 2023), and achieve superior intervention outcomes (Tian et al., 2024; Miller, 2020). Mind-body exercise places greater emphasis on emotional and conscious regulation and the enhancement of neural network function and plasticity compared to aerobic and resistance exercise. Aerobic exercise has limited influence on deep autonomic regulation and resistance exercise exerts relatively modest effects on neural plasticity (Miller et al., 2021). Mind-body exercise intervention has been demonstrated to yield substantial benefits for cognitive function (Wang & Lyu, 2024) and mental health, as well as reduce symptoms of depression and anxiety.

Recent several systematic reviews and meta-analyses have examined the effectiveness of mind-body exercise interventions for depression and anxiety. Tian et al. (2024) found that yoga is the most effective mind-body exercise modality for alleviating depressive symptoms, followed by qigong and tai chi. Lin et al. (2024) showed in their systematic review that mind-body exercises can effectively reduce anxiety, and highlighted exercise duration per session as a moderating factor, with 60-min sessions demonstrating optimal effectiveness. Zou et al.’s (2018b) systematic review and meta-analysis also reported that mind-body exercise reduces depression and anxiety; however, subgroup analyses comparing active and passive control conditions revealed no significant advantage of mind-body exercises over passive conditions. While the overall effectiveness of mind-body exercises for alleviating depression and anxiety is positive and significant, consensus regarding specific exercise-related factors influencing exercise outcomes remains unclear or contentious. Additionally, different exercise parameters, such as exercise type, duration, frequency, and cycle, may selectively influence the effects on depression and anxiety (Dong et al., 2024). This study builds upon previous research and employs meta-analysis to systematically evaluate the effectiveness of mind-body exercises on anxiety and depression, further clarifying differences related to exercise type, session duration, frequency, exercise cycle, and baseline levels of depression and anxiety, in order to provide evidence-based recommendations for clinical mind-body exercise programs.

Drawing from prior studies, we observed ongoing debate surrounding the effectiveness of mind-body exercises in alleviating symptoms of depression and anxiety. According to Miller (2020), mind-body exercise interventions demonstrate significantly greater effectiveness compared to aerobic and resistance exercises, whereas Lin et al. (2024) reported no significant differences in the anti-anxiety effects between mind-body exercises and other exercise modalities. Consequently, this study adopts an evidence-based medicine approach to systematically assess the effectiveness of mind-body exercises in mitigating symptoms of depression and anxiety. Furthermore, it examines the varying impacts of exercise type, duration, frequency, and cycle on these symptoms, with the objective of delineating the effectiveness and optimal dose-response relationship of mind-body exercise interventions. This study provides robust evidence-based support for leveraging mind-body exercises as an intervention in alleviating symptoms of depression and anxiety. This study primarily targets researchers in the field of sports psychology and aims to provide up-to-date evidence on the use of mind-body exercise in addressing depression and anxiety.

## Methods

### Literature retrieval strategy

This study adheres to the guidelines outlined by the Preferred Reporting Items for Systematic Reviews and Meta-Analyses (PRISMA) (Page et al., 2021) in order to ensure a high degree of methodological transparency and rigor. The study is registered on the PROSPERO international systematic review platform (https://www.crd.york.ac.uk/prospero/) under registration number CRD42024613769, thereby reinforcing its credibility and adherence to systematic review protocols.

The researcher, Zheng Ye, performed a comprehensive search across multiple databases, including Web of Science, PubMed, Cochrane Library, Embase, China National Knowledge Infrastructure (CNKI), WANFANG DATA, and the VIP Chinese Science and Technology Journal Database (VIP database), to identify randomized controlled trials (RCTs) that investigated the effects of mind-body exercise interventions on depression and anxiety symptoms. The search cycle for all selected databases spanned from their respective inception dates to July 1, 2024.

The search strategy was designed using a combination of subject terms and free-text terms. Chinese search terms included: 抑郁, 焦虑, 情绪, 身心运动, 太极, 太极拳, 气功, 八段锦, 易筋经, 五禽戏, 瑜伽等. English search terms included: Depression, Depressive Disorder, Anxiety, Affective, Mood, Emotion, Taiji, Taijiquan, Tai Chi, Tai Chi Chua, Tai ji, Tai ji quan, Taichi, Mind body, Yoga, Qigong, Qi gong, Yijinjing, Yi Jin Jing, Baduanjin, Ba duan jin, Wuqinxi, Wu Qin Xi.

### Data extraction

Two researchers (Zheng Ye and Zhihui Xu) independently conducted a comprehensive review of the literature, systematically extracted relevant data, and performed statistical analyses based on the predefined inclusion and exclusion criteria. The results were cross-verified to ensure accuracy. In the event of any discrepancies or disagreements, a third researcher (Xing Wang) was consulted to mediate the discussion and facilitate the resolution of any conflicts, ensuring a consensus was ultimately reached. Relevant data were systematically extracted using a pre-established form, which captured essential details such as the author(s), publication year, fundamental characteristics of the study participants, the specific exercise measures implemented for the exercise group, and the defined outcome indicators.

### Inclusion and exclusion criteria

To minimize heterogeneity and improve internal validity, only randomized controlled trials comparing mind-body exercise with passive control groups (*e.g*., waitlist, observation, or no-intervention groups) were included. Studies that compared mind-body exercise with active control groups (*e.g*., aerobic exercise, stretching, or other forms of physical activity) were excluded due to the potential introduction of confounding factors that could obscure the independent effects of mind-body exercise.

The inclusion criteria were defined as follows:

(1) Study population: Individuals diagnosed with depression or anxiety according to the International Classification of Diseases (ICD) or the Diagnostic and Statistical Manual of Mental Disorders (DSM). No restrictions were imposed regarding ethnicity or gender.
(2) Experimental group: Participants who engaged in a single mind-body exercise, such as tai chi, qigong (including Baduanjin, Wuqinxi, and Mawangdui Daoyin), or yoga.
(3) Control group: Participants who did not engage in any exercise intervention or other forms of therapeutic intervention.
(4) Primary outcome measures: Validated psychological assessment instruments, such as the Hamilton Depression Rating Scale (HAMD), Self-Rating Depression Scale (SDS), Symptom Checklist 90 (SCL-90), Depression Anxiety Stress Scales (DASS), Center for Epidemiological Studies Depression Scale (CES-D), Self-Rating Anxiety Scale (SAS), State Anxiety Inventory (STAI), and Hospital Anxiety and Depression Scale (HADS), with scores employed as study endpoints.

The exclusion criteria were defined as follows:

(1) Participants: Individuals who were on antidepressants, anti-anxiety medications, or other psychotropic drugs. Studies in which the control group engaged in physical exercise, particularly where the experimental group performed mind-body exercises while the control group participated in alternative forms of exercise, were also excluded.
(2) Interventions: Studies employing combined approaches, such as exercise paired with medication or cognitive training, were excluded. Additionally, studies in which both experimental and control groups received treatments such as medication or psychological interventions, as well as those utilizing multiple exercise or treatment modalities, were omitted.
(3) Outcome measures: Studies lacking pre- and post-intervention assessments of depression or anxiety symptoms were excluded.

### Literature screening and data extraction

A systematic literature search was performed across seven databases, and the retrieved records were imported in bulk into EndNote X9 reference management software. Following the import, duplicate records were identified and removed using EndNote’s built-in deduplication feature. Titles and abstracts of the remaining studies were reviewed to conduct an initial screening, while full-text reviews were subsequently undertaken to identify eligible studies based on the predefined inclusion and exclusion criteria. Extracted data included the first author’s name, publication year, country, study population characteristics, intervention details, and outcome measures.

In cases where a study featured multiple experimental groups and a single control group, only the experimental group performing mind-body exercises was selected for inclusion. Furthermore, when studies had multiple mind-body exercise groups or reported outcomes for both anxiety and depression, the data were partitioned and analyzed separately. For studies employing multiple measurement tools, the tool most frequently cited within the study was prioritized for inclusion in the analysis.

### Quality evaluation of included literature

The Physiotherapy Evidence Database (PEDro) scale (Verhagen et al., 1998) was employed to assess the methodological rigor of the included studies. This scale was comprised of 11 distinct criteria: eligibility criteria, randomization, concealed allocation, baseline comparability, blinding of participants, blinding of therapists, blinding of outcome assessors, retention rate exceeding 85%, intention-to-treat analysis, between-group comparisons, and point estimates with associated variability measures. Eligibility criteria were not scored. Each explicitly satisfied criterion earned one point, with a maximum score of 10, and was classified as follows: 9–10 indicating excellent quality, 6–8 indicating good quality, 4–5 indicating fair quality, and below 4 indicating poor quality.

The Grading of Recommendations, Assessment, Development, and Evaluations (GRADE) profile tool (Page et al., 2021) was employed to systematically assess the quality of evidence. This evaluation considered five key factors that could lead to downgrading: publication bias, inconsistency, imprecision, indirectness, and study limitations. Evidence quality was classified into four levels: high (no downgrade), moderate (downgraded by one level), low (downgraded by two levels), and very low (downgraded by three levels). Two researchers conducted independent assessments, with discrepancies resolved through consultation with a third researcher to ensure consensus.

### Data analysis

In this meta-analysis, Hedges’s g was used as the effect size metric to compare the standardized mean differences between the exercise and control groups. Hedges’s g is an unbiased estimator that adjusts for small sample sizes and is commonly employed in meta-analyses of continuous outcomes. The effect size was calculated as the difference between the post-intervention mean values of the exercise and control groups, divided by the pooled standard deviation, and adjusted using a correction factor for small sample bias. Ninety-five percent confidence intervals (CIs) were computed for all effect sizes to estimate the precision of the effect size estimates. The 95% CI provides a range of values within which the true effect size is expected to lie with 95% certainty; a CI that does not include zero indicates a statistically significant difference between groups.

Stata 17 software was utilized to perform a comprehensive meta-analysis, focusing on the sample sizes and the mean and standard deviation of improvement scores before and after the intervention for all included outcome measures. Since all outcome measures were continuous variables, Hedges’s g was selected as the effect size metric. A 95% CI that did not include 0 was considered indicative of a statistically significant difference. Effect sizes were categorized as follows: <0.2 (small), 0.20–0.50 < (small-to-moderate), 0.50–0.80 < (moderate), and ≥ 0.8 (large). All reported effect sizes included point estimates and 95% CIs, with statistical significance established at *P* < 0.05.

This study conducted effect size pooling, subgroup analyses, heterogeneity assessments, and sensitivity evaluations for the included studies. Following the effect size pooling, subgroup analyses were performed to explore potential sources of heterogeneity by stratifying the studies based on exercise type (*e.g*., qigong, tai chi, yoga), session duration, weekly frequency, exercise cycle, and baseline levels of depression and anxiety. Heterogeneity was quantified using the *I*^2^ statistic alongside the corresponding *P*-value. An *I*^2^ ≤ 25% indicated no heterogeneity, 25% < *I*^2^ ≤ 50% indicated low heterogeneity, 50% < *I*^2^ ≤ 75% indicated moderate heterogeneity, and *I*^2^ > 75% indicated high heterogeneity. When *I*^2^ exceeded 70%, sensitivity analysis was conducted by sequentially removing outliers to assess the robustness of the pooled results (Chen, Du & Zhu, 2025). A random-effects model was applied that acknowledged heterogeneity among the included studies. When *I*^2^ < 50%, potential sources of heterogeneity were further investigated. In addition, sensitivity analyses were conducted by iteratively excluding individual studies to examine whether the pooled effect size changed substantially, thereby testing the robustness of the findings. Statistical significance for the meta-analysis results was determined at *P* < 0.05. To evaluate publication bias for the outcome measures, a funnel plot was generated with Stata 17.0 software.

## Results

### Literature retrieval results

**Figure 1:**
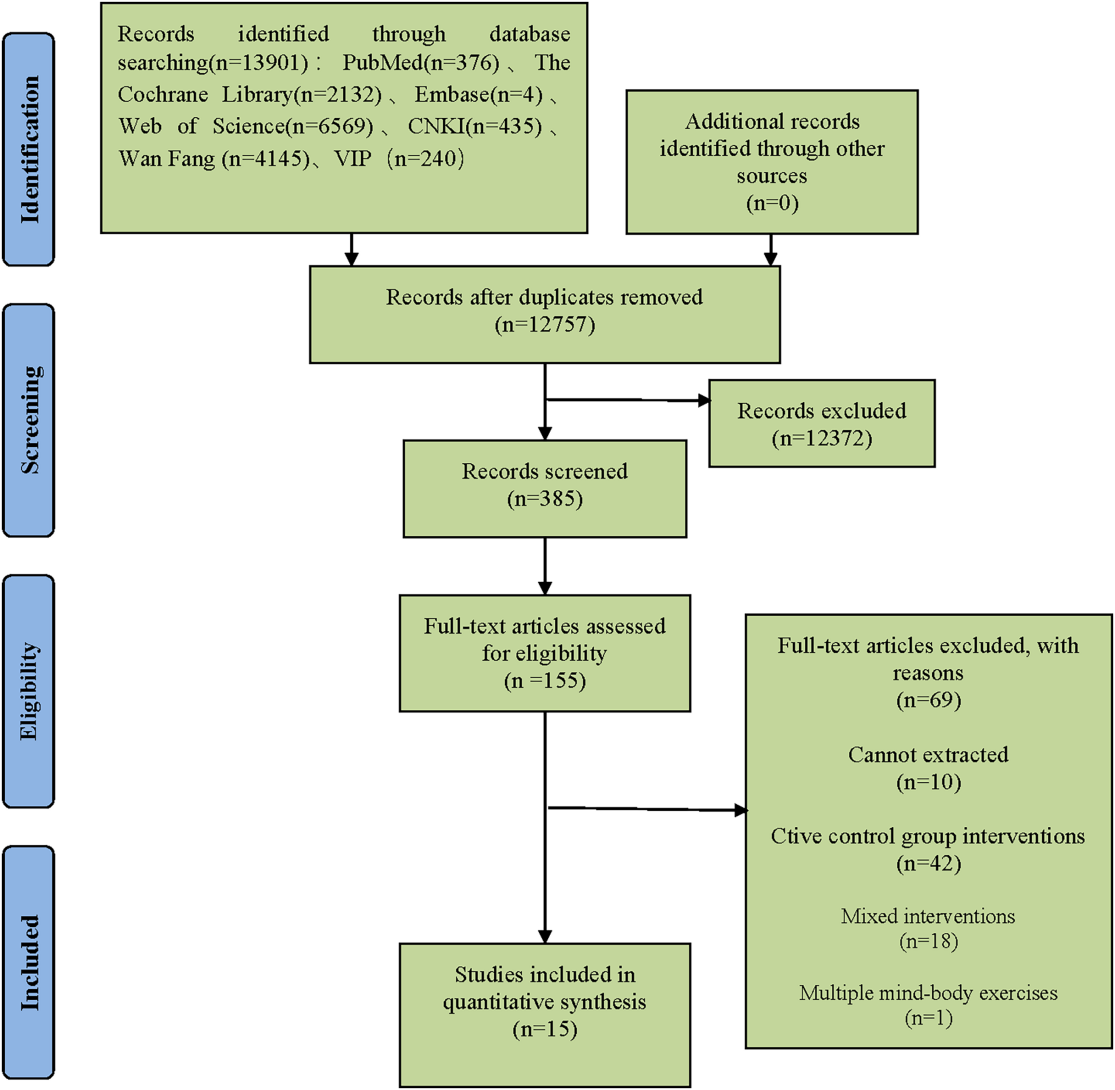
Literature screening process.

A total of 13,891 records were initially identified through searches across seven major databases, including Web of Science, PubMed, Cochrane Library, Embase, CNKI, WANFANG DATA, and the VIP database. After duplicates and irrelevant records were removed, 385 articles underwent full-text screening based on the predefined inclusion criteria. Subsequently, 155 studies were deemed eligible for detailed review. Of these, 69 were excluded due to non-conformance with inclusion criteria, 10 lacked extractable data, and 61 involved non-conforming interventions. Ultimately, 15 studies were included in the analysis, comprised of nine Chinese and six English publications. The selection process is illustrated in Fig. 1.

### Basic features of included studies

A total of 15 studies, encompassing 1,351 participants, were included in this analysis, with 787 participants assigned to the experimental group and 564 to the control group. All participants had been diagnosed with depression or anxiety. The studies were published between 2010 and 2024, providing a comprehensive temporal coverage of recent research. Among these, nine studies examined both depression and anxiety, five focused exclusively on depression, and one focused exclusively on anxiety.

In all included studies, participants in the experimental group engaged in mind-body exercises, including qigong, tai chi, and yoga, while the control group abstained from any form of exercise intervention. Neither group received any additional therapeutic measures, ensuring the isolation of exercise effects as the primary variable. The duration of individual exercise sessions varied between 20 and 90 min, while exercise cycles spanned 8 to 24 weeks. The frequency of exercise ranged from once weekly to daily sessions, reflecting diverse program structures across the studies. The mean age of participants ranged from 21.1 ± 1.4 to 66.64 ± 7.33 years, highlighting the broad demographic range represented in the included studies. Detailed information can be found in Table 1.

**Table 1:** Basic characteristics of the literature.

Study	Country/Area	Sample size (T/C)	Age (years) T/C	Exercise type	Session duration (min)	Exercise period	Frequency (times/week)	Study population	Participant characteristics	Baseline depression/anxiety severity (T)	Measurements
Ma et al. (2010)	China	50/50	49.08 ± 2.76/49.30 ± 2.59	Qigong	45	3 Months	7	Middle-aged and older women	Residents of Xishandao Community, Tangshan City	25.14 ± 2.95 (High)	CES-D
Ma et al. (2011)	China	49/50	46.89 ± 2.69/46.92 ± 2.31	Qigong	90	3 Months	7	Perimenopausal women	Residents of Wenhua Road Community, Tangshan City	25.10 ± 2.89 (High)	CES-D
Chow, Dorcas & Siu (2012)	China, H K	34/31	44.2 ± 11.03/44.66 ± 11.86	Qigong	90	8 Week	2	Adults interested in Qigong	Hong Kong residents	6.29 ± 5.33 (Normal)/8.24 ± 6.53 (Mild)	DASS
Field et al. (2013)	USA	40/39	24.9 ± 5.2/24.5 ± 5.02	Yoga	20	12 Week	1	Pregnant women	Hispanic or African American women in the United States	30.0 ± 10.2 (High)/55.0 ± 8.8 (High)	CES-D/STAI
Chan et al. (2013)	China, H K	72/65	42.5 ± 6.4/42.5 ± 6.4	Qigong	30	17 Week	7	Patients with chronic fatigue syndrome	Community residents in Hong Kong	9.1 ± 2.0 (Mild)/11.0 ± 2.1 (Moderate)	HADS
Buttner et al. (2015)	USA	23/27	29.81 ± 5.17/32.45 ± 4.78	Yoga	60	8 Week	2	Women with postpartum depression	English-speaking women aged 18–45	17.33 ± 5.10 (Moderate)	HAMD
Gao (2016)	China	149/37	66.64 ± 7.33/64.75 ± 5.27	Qigong	60	24 Week	≥5	Older adults	Older adults living in Xishan District, Wuxi City	36.76 ± 6.14 (Normal)/36.86 ± 6.50 (Normal)	SDS/SAS
Liu (2016)	China	32/31	66.3 ± 2.7/65.8 ± 3.2	Taijiquan	60	16 Week	5	Healthy older women	Recruited from elderly care institutions, parks, and communities	1.80 ± 0.54 (Mild)/1.56 ± 0.37 (Mild)	SCL-90
Fu et al. (2016)	China	100/100	28.30 ± 7.83/27.99 ± 8.17	Qigong	30	5 Months	7	Patients in drug rehabilitation programs	Female inmates in a drug rehabilitation center	56.00 ± 9.95 (Mild)/51.72 ± 9.84 (High)	SDS/SAS
Ma, Wang & Xi (2016)	China	38/40	60.9 ± 5.1/60.2 ± 4.1	Qigong	60	20 Week	3	Middle-aged and older women	Older women residing in community settings in Shanghai	2.12 ± 2.65 (Moderate)/38.72 ± 7.26 (Normal)	POMS/SAS
Cheng et al. (2016)	China	15/15	21.1 ± 1.4/21.0 ± 1.6	Qigong	40–60	12 Week	3	University students with mild depression	University student volunteers	16.2 ± 2.3 (Mild)	HAMD
Telles et al. (2016)	India	20/20	36.10 ± 7.33/37.40 ± 4.85	Yoga	60	12 Week	7	Individuals with state anxiety	Patients with degenerative disc disease	48.40 ± 12.63 (High)	STAI
Zhao et al. (2017)	China	30/30	51.38 ± 14.83/53.85 ± 11.69	Taijiquan	30	8 Week	5	Patients with post-stroke depression	Patients with post-stroke depression attending a hospital	24.70 ± 3.55 (High)	HAMD
Hua & Sun (2021)	China	60/20	20.38 ± 0.804/21.15 ± 1.040	Taijiquan	80	12 Week	1	University students	Non-physical education major university students	6.27 ± 5.11 (Normal)/8.30 ± 5.62 (Mild)	DASS
Hua & Sun (2021) (1)	China	66/20	20.29 ± 0.837/21.15 ± 1.040	Yoga	80	12 Week	1	University students	Non-physical education major university students	6.97 ± 4.71 (Normal)/9.18 ± 5.54 (Mild)	DASS
Zhang et al. (2023a)	China	9/9	22.50 ± 5.95/22.50 ± 5.95	Taijiquan	60	8 Week	5	University students	Depressed and anxious university students at Beijing Normal University	69.29 ± 12.16 (Moderate)/56.94 ± 9.23 (Mild)	SDS/SAS

**Note:**

The assessment of baseline depression/anxiety severity was based on the measurement instruments listed in the “Measurements” column, with severity levels corresponding to the scoring criteria of each respective scale. CES-D, Center for Epidemiological Studies Depression Scale; STAI, State-Trait Anxiety Inventory; HADS, Hospital Anxiety and Depression Scale; HAMD, Hamilton Depression Rating Scale; SDS, Self-Rating Depression Scale; SAS, Self-Rating Anxiety Scale; SCL-90, Symptom Checklist-90; POMS, Profile of Mood States; HAMA, Hamilton Anxiety Rating Scale; DASS, Depression Anxiety Stress Scales.

### Quality evaluation of the included literature

All included studies were RCTs, with PEDro scale scores ranging from 6 to 9 and an average score of 6.33, which reflected the overall good methodological quality of the research. Among the included studies, one implemented participant, therapist, and outcome assessor blinding, while another applied allocation concealment, which demonstrated adherence to rigorous methodological practices. All studies achieved random allocation and reported participant retention rates exceeding 85%, underscoring the reliability of the research findings. For further details, refer to Table 2.

**Table 2:** Study quality assessment of all selected trials.

Studies	Item 1	Item 2	Item 3	Item 4	Item 5	Item 6	Item 7	Item 8	Item 9	Item 10	Item 11	Score
Ma et al. (2010)	√	√		√				√	√	√	√	6
Ma et al. (2011)	√	√		√				√	√	√	√	6
Chow, Dorcas & Siu (2012)	√	√		√				√	√	√	√	6
Field et al. (2013)	√	√		√				√	√	√	√	6
Chan et al. (2013)	√	√		√				√	√	√	√	6
Buttner et al. (2015)	√	√		√				√	√	√	√	6
Gao (2016)	√	√		√	√	√	√	√	√	√	√	9
Liu (2016)	√	√		√				√	√	√	√	6
Fu et al. (2016)	√	√		√				√	√	√	√	6
Ma, Wang & Xi (2016)	√	√		√				√	√	√	√	6
Cheng et al. (2016)	√	√		√				√	√	√	√	6
Telles et al. (2016)	√	√	√	√				√	√	√	√	7
Zhao et al. (2017)	√	√		√				√	√	√	√	6
Hua & Sun (2021)	√	√		√				√	√	√	√	6
Zhang et al. (2023a)	√	√	√	√				√	√	√	√	7

**Note:**

Item 1, specified eligibility criteria; Item 2, random allocation; Item 3, concealed allocation; Item 4, baseline equivalence; Item 5, Research object blinding; Item 6, therapist blinding; Item 7, assessor blinding; Item 8, retention rate ≥85%; Item 9, intention-to-treat analysis for missing data; Item 10, between-group statistical comparison; and Item 11, point measure/measure of variability ≥ one key outcome.

### Meta-analysis and subgroup analysis

#### Meta-analysis of mind-body exercise interventions for depression

**Figure 2:**
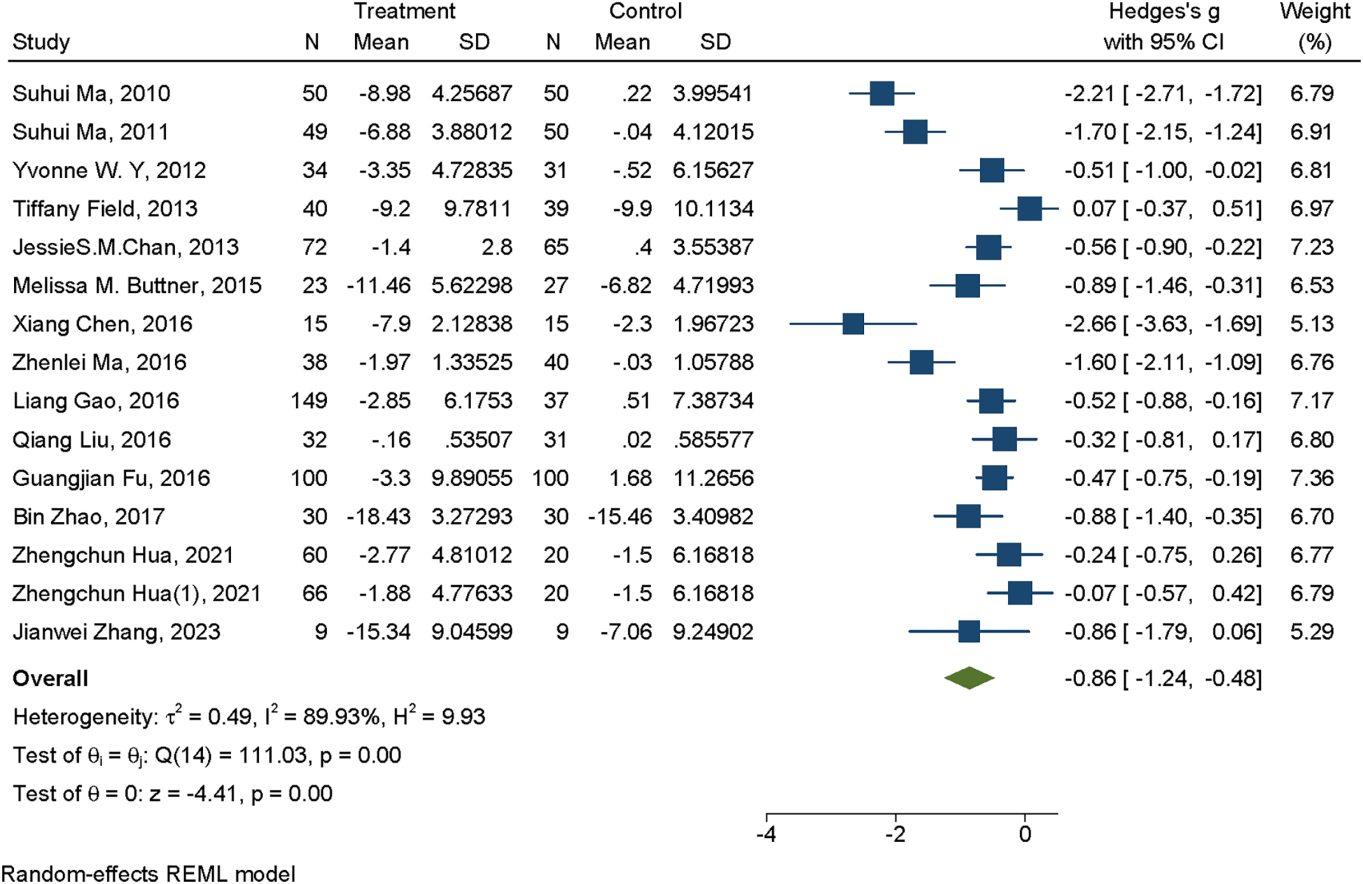
Forest plot of mind-body exercise interventions for depression. Studies: Ma et al. (2010), Ma et al. (2011), Chow, Dorcas & Siu (2012), Field et al. (2013), Chan et al. (2013), Buttner et al. (2015), Gao (2016), Liu (2016), Fu et al. (2016), Ma, Wang & Xi (2016), Cheng et al. (2016), Telles et al. (2016), Zhao et al. (2017), Hua & Sun (2021), Zhang et al. (2023a).

A total of 14 studies investigated the effects of mind-body exercise on depressive symptoms. In the forest plot, the effect size (Hedges’s *g*) and corresponding 95% CI for the impact of mind-body exercise on depression were presented. The heterogeneity analysis indicated substantial heterogeneity among studies (*I*^2^ = 89.98%, *P* < 0.01). In the plot, each square represents the standardized mean difference of an individual study, with the size of the square reflecting the study’s weight in the meta-analysis. The horizontal line denotes the 95% CI for the estimated effect size of each study. The diamond at the bottom of the figure represents the pooled overall effect size and its 95% CI. Negative effect sizes indicate that depressive symptoms in the exercise group were significantly lower than those in the control group. The meta-analysis yielded a moderately large and statistically significant effect (Hedges’s *g* = –0.86, 95% CI [–1.24 to –0.48], *P* < 0.01), suggesting that mind-body exercise has a beneficial effect in alleviating depressive symptoms (see Fig. 2).

#### Meta-analysis of mind-body exercise interventions for anxiety

**Figure 3:**
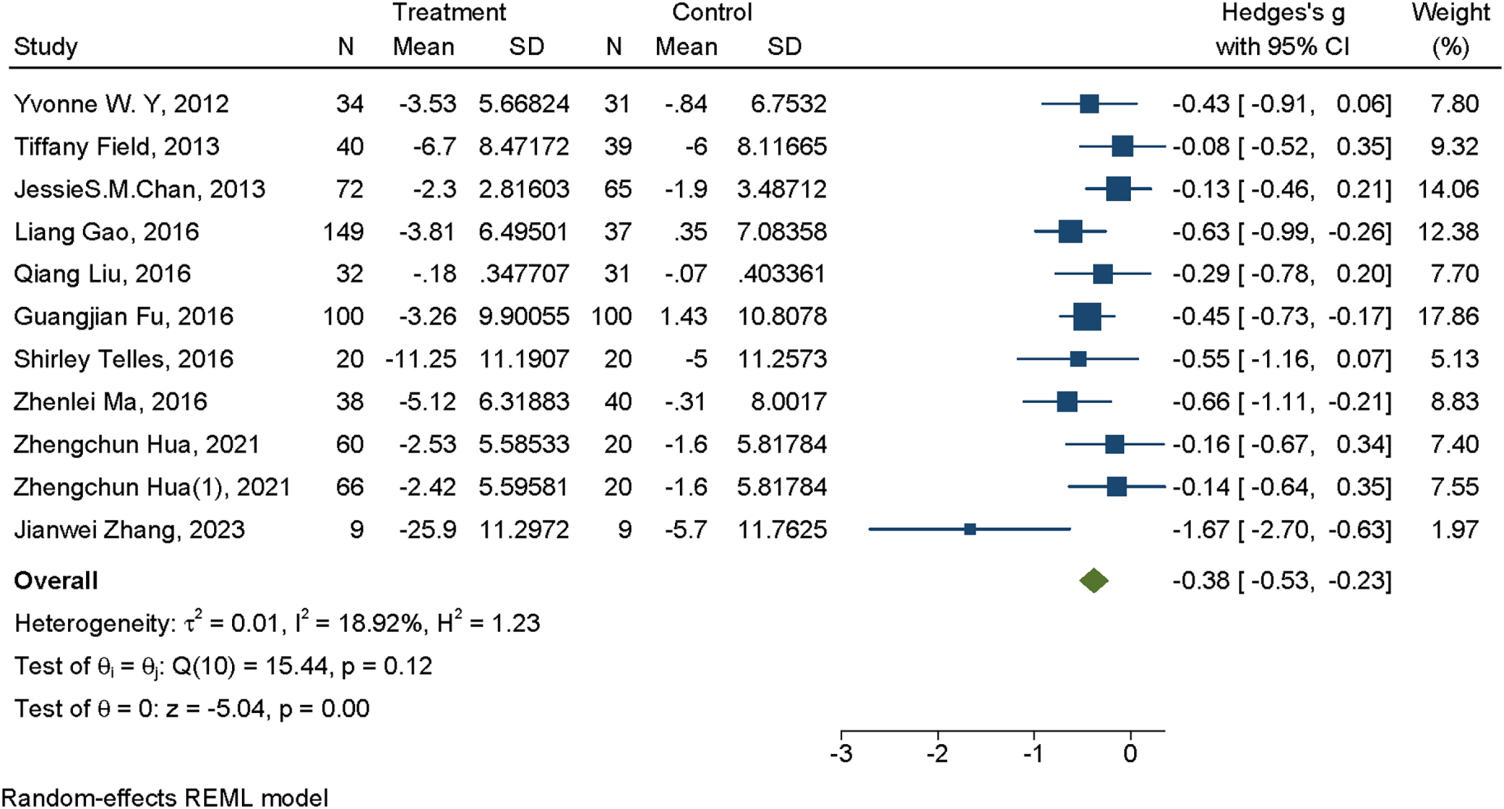
Forest plot of mind-body exercise interventions for anxiety. Studies: Chow, Dorcas & Siu (2012), Field et al. (2013), Chan et al. (2013), Gao (2016), Liu (2016), Fu et al. (2016), Ma, Wang & Xi (2016), Cheng et al. (2016), Telles et al. (2016), Hua & Sun (2021), Zhang et al. (2023a).

A total of 10 studies examined the effects of mind-body exercise on anxiety symptoms. In the forest plot, the standardized effect sizes (Hedges’s *g*) and their corresponding 95% CI for the impact of mind-body exercise on anxiety were presented. The heterogeneity test indicated that there was no significant heterogeneity among the studies (*I*^2^ = 18.92%, *P* < 0.01). In the plot, each square represents an individual study, with the size of the square reflecting its statistical weight. The horizontal line denotes the 95% CI, and the diamond at the bottom represents the pooled effect estimate and its CI. Negative effect sizes indicate that anxiety symptoms improved more in the exercise group than in the control group. The meta-analysis yielded a statistically significant small-to-moderate effect (Hedges’s *g* = –0.38, 95% CI [–0.53 to –0.23], *P* < 0.01), suggesting that mind-body exercise has a beneficial effect in reducing anxiety levels (see Fig. 3).

#### Subgroup analysis

To explore potential sources of heterogeneity, subgroup analyses were conducted based on exercise type, session duration, frequency, exercise cycle, and baseline severity of depression or anxiety.

In studies targeting depression, exercise type was categorized into Qigong, Tai Chi, and Yoga. Session duration was grouped as 31–60 min, ≤30 min, and >60 min. Exercise cycle was divided into 9–12 weeks, ≤8 weeks, and >12 weeks. Exercise frequency was categorized as once per week, twice per week, three times per week, and ≥ four times per week. Baseline depression severity was classified as high, moderate, low, or normal.

In studies targeting anxiety, exercise type was also categorized into Qigong, Tai Chi, and Yoga. Session duration was grouped as 31–60 min, ≤30 min, and >60 min. Exercise cycle was categorized as 9–16 weeks, ≤8 weeks, and >16 weeks. Exercise frequency was grouped as ≥ four times per week or < four times per week. Baseline anxiety severity was classified as high, moderate, low, or normal.

##### Subgroup analysis of mind-body exercise interventions for depression

The results indicated that exercise type (*Q* = 6.04, *P* = 0.049), session duration (*Q* = 10.47, *P* = 0.005), frequency (*Q* = 22.07, *P* < 0.001), and baseline depression severity (*Q* = 9.94, *P* = 0.019) were significant moderators, whereas exercise cycle (*Q* = 0.67, *P* = 0.714) was not.

In research on depression, subgroup analyses were performed to assess the impact of different exercise modalities, including Tai Chi, Yoga, and Qigong. The findings revealed that Tai Chi (Hedges’s *g* = −0.517, 95% CI [−0.859 to −0.174], *P* = 0.003) and Qigong (Hedges’s *g* = −1.228, 95% CI [−1.805 to −0.650], *P* < 0.001) significantly alleviated depressive symptoms. When stratified by single-session duration, exercises lasting 31–60 min (Hedges’s *g* = −1.315, 95% CI [−1.874 to −0.755], *P* < 0.001) and those ≤30 min (Hedges’s *g* = −0.451, 95% CI [−0.793 to −0.109], *P* = 0.010) demonstrated significant benefits, emphasizing the importance of limiting individual sessions to 60 min or less. Analysis by exercise cycle suggested consistent effectiveness across all groups. Furthermore, subgroup analyses based on exercise frequency indicated that engaging in exercise twice per week (Hedges’s *g* = −0.670, 95% CI [−1.042 to −0.297], *P* < 0.001), three times per week (Hedges’s *g* = −2.045, 95% CI [−3.070 to −1.020], *P* < 0.001), and four times per week (Hedges’s *g* = −0.930, 95% CI [−1.042 to −0.458], *P* < 0.001) produced significant reductions in depressive symptoms. Subgroup analyses based on baseline depression severity showed that participants with high (Hedges’s *g* = –1.176, 95% CI [–2.157 to –0.195], *P* = 0.019), low (Hedges’s *g* = –1.174, 95% CI [–1.701 to –0.648], *P* < 0.001), and normal (Hedges’s *g* = –0.372, 95% CI [–0.596 to –0.147], *P* < 0.001) levels of depression all experienced improvement. For further details, refer to Table 3.

**Table 3:** Subgroup analysis for depression.

	Group	Number of studies	*I*^2^/%	Hedges’s g (95% CI)	*P* value	Q_b	*P* > Q_b
Exercise type	Qigong	8	93.12	−1.228 [−1.805 to −0.650]	<0.001	6.04	0.049
	Taijiquan	4	30.65	−0.517 [−0.859 to −0.174]	0.003		
	Yoga	3	73.81	−0.273 [−0.837 to 0.291]	0.343		
Session duration (min)	31–60	8	88.43	−1.315 [−1.874 to −0.755]	<0.001	10.47	0.005
	≤30	4	68.85	−0.451 [−0.793 to −0.109]	0.01		
	>60	3	<0.01	−0.279 [−0.565 to 0.007]	0.056		
Exercise period (weeks)	9–12 week	6	94.94	−1.104 [−2.044 to −0.164]	0.021	0.67	0.714
	≤8 week	4	<0.01	−0.751 [−1.039 to −0.463]	<0.001		
	>12 week	5	83.04	−0.674 [−1.087 to −0.261]	0.001		
Frequency (times/week)	1	3	<0.01	−0.068 [−0.342 to 0.207]	0.63	22.07	0.000
	2	2	<0.01	−0.670 [−1.042 to −0.297]	<0.001		
	3	2	72.31	−2.045 [−3.070 to −1.020]	<0.001		
	≥4	8	89.55	−0.930 [−1.402 to −0.458]	<0.001		
Baseline depression severity	High	4	94.11	−1.176 [−2.157 to −0.195]	0.019	9.94	0.019
	Mild	4	95.24	−0.926 [−1.906 to 0.054]	0.064		
	Moderate	3	50.71	−1.174 [−1.701 to −0.648]	<0.001		
	Normal	4	0.00	−0.372 [−0.596 to −0.147]	0.001		

##### Subgroup analysis of mind-body exercise interventions for anxiety

The results showed that exercise type (*Q* = 2.02, *P* = 0.365), session duration (*Q* = 1.79, *P* = 0.409), exercise cycle (*Q* = 3.01, *P* = 0.222), frequency (*Q* = 0.88, *P* = 0.349), and baseline anxiety severity (*Q* = 5.59, *P* = 0.134) were not significant moderators.

In investigations on anxiety, subgroup analyses were conducted to evaluate the effects of various exercise modalities, including Tai Chi, Yoga, and Qigong. Among these, Qigong demonstrated a significant reduction in anxiety symptoms (Hedges’s *g* = −0.439, 95% CI [−0.634 to −0.245], *P* < 0.001). When stratified by single-session duration, exercises lasting 31–60 min were associated with significant improvements in anxiety (Hedges’s *g* = −0.507, 95% CI [−0.780 to −0.234], *P* < 0.001). Analysis based on exercise cycle suggested that programs lasting longer than 16 weeks yielded substantial benefits (Hedges’s *g* = −0.445, 95% CI [−0.678 to −0.212], *P* < 0.001). Furthermore, subgroup analysis by exercise frequency indicated effectiveness across all frequency categories. Subgroup analyses based on baseline anxiety severity showed improvements among participants with high (Hedges’s *g* = –0.362, 95% CI [–0.613 to –0.111], *P* = 0.005), moderate (Hedges’s *g* = –0.334, 95% CI [–0.574 to –0.094], *P* = 0.006), and normal (Hedges’s *g* = –0.639, 95% CI [–0.923 to –0.356], *P* < 0.001) levels of anxiety. For further details, refer to Table 4.

**Table 4:** Subgroup analysis for anxiety.

	Group	Number of studies	*I*^2^/%	Hedges’s g MD (95% CI)	*P* value	Q_b	*P* > Q_b
Exercise type	Qigong	5	27.96	−0.439 [−0.634 to −0.245]	<0.001	2.02	0.365
	Taijiquan	3	80.28	−0.593 [−1.407 to 0.221]	0.153		
	Yoga	3	0.00	−0.205 [−0.495 to 0.084]	0.165		
Session duration (min)	31–60	6	47.68	−0.507 [−0.780 to −0.234]	<0.001	1.79	0.409
	≤30	2	48.06	−0.307 [−0.658 to −0.044]	0.087		
	>60	3	0.00	−0.248 [−0.534 to 0.038]	0.089		
Exercise period (weeks)	9–16 week	5	0.00	−0.214 [−0.438 to −0.009]	0.06	3.01	0.222
	≤8 week	2	77.86	−0.961 [−2.164 to 0.243]	0.118		
	>16 week	4	43.75	−0.445 [−0.678 to −0.212]	<0.001		
Frequency (times/week)	≥4	6	36.43	−0.450 [−0.672 to −0.229]	<0.001	0.88	0.349
	<4	5	11.79	−0.299 [−0.524 to −0.074]	0.009		
Baseline anxiety severity	High	3	15.45	−0.362 [−0.613 to −0.111]	0.005	5.59	0.134
	Mild	5	0.00	−0.334 [−0.574 to −0.094]	0.006		
	Moderate	1	.	−0.126 [−0.460 to 0.208]	0.459		
	Normal	2	0.00	−0.639 [−0.923 to −0.356]	<0.001		

#### Sensitivity analysis

**Figure 4:**
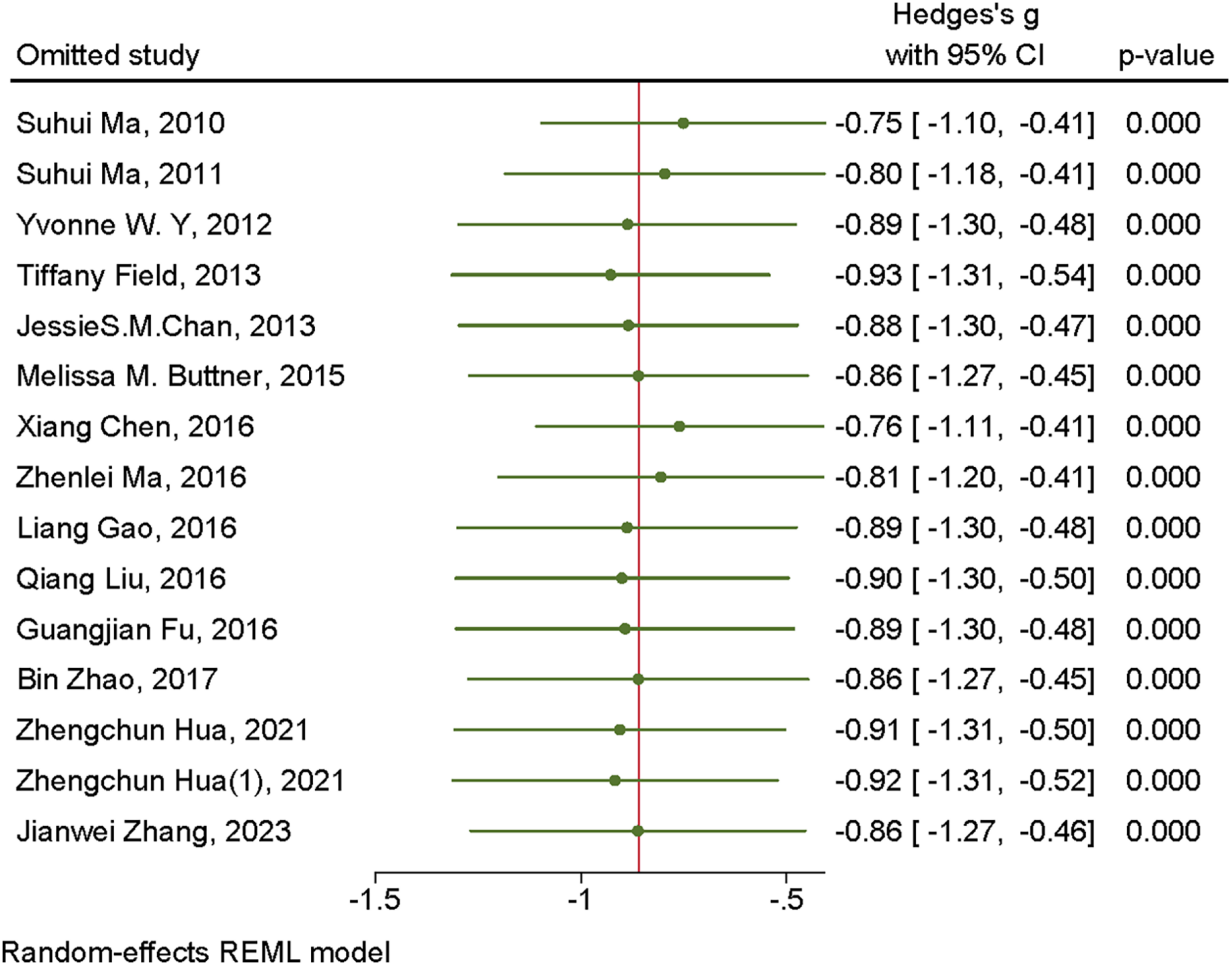
Sensitivity analysis of mind-body exercises for depression. Studies: Ma et al. (2010), Ma et al. (2011), Chow, Dorcas & Siu (2012), Field et al. (2013), Chan et al. (2013), Buttner et al. (2015), Gao (2016), Liu (2016), Fu et al. (2016), Ma, Wang & Xi (2016), Cheng et al. (2016), Zhao et al. (2017), Hua & Sun (2021), Zhang et al. (2023a).

**Figure 5:**
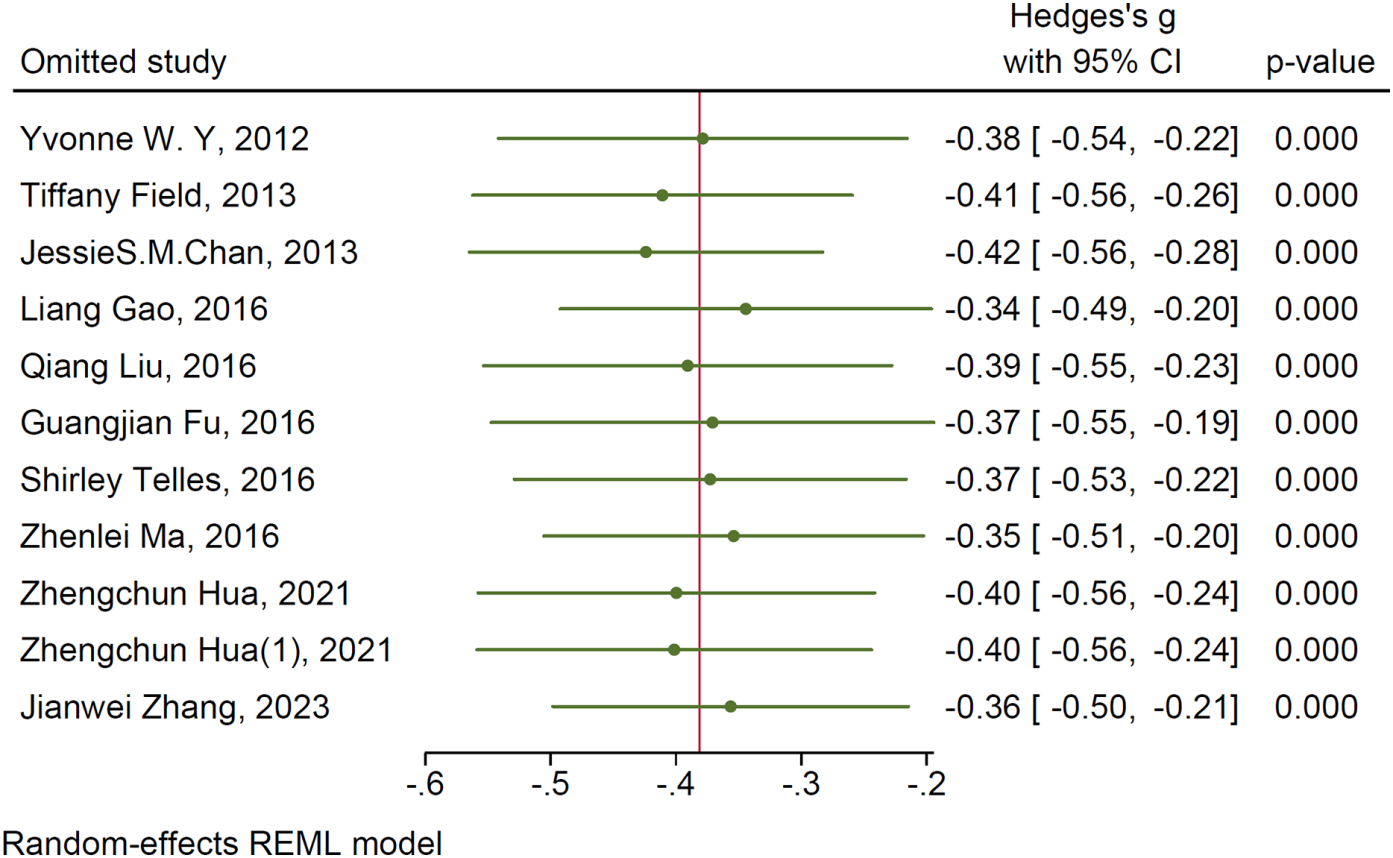
Sensitivity analysis of mind-body exercises for anxiety. Studies: Chow, Dorcas & Siu (2012), Field et al. (2013), Chan et al. (2013), Gao (2016), Liu (2016), Fu et al. (2016), Telles et al. (2016), Ma, Wang & Xi (2016), Cheng et al. (2016), Zhao et al. (2017), Hua & Sun (2021), Zhang et al. (2023a).

To further investigate the sources of heterogeneity, sensitivity analyses were conducted to evaluate whether individual studies contributed to the observed heterogeneity. The findings revealed that sequentially excluding individual studies did not lead to substantial changes in the effect size, suggesting that the results were robust and relatively stable. Refer to Figs. 4 and 5 for detailed visualization.

### Publication bias test

**Figure 6:**
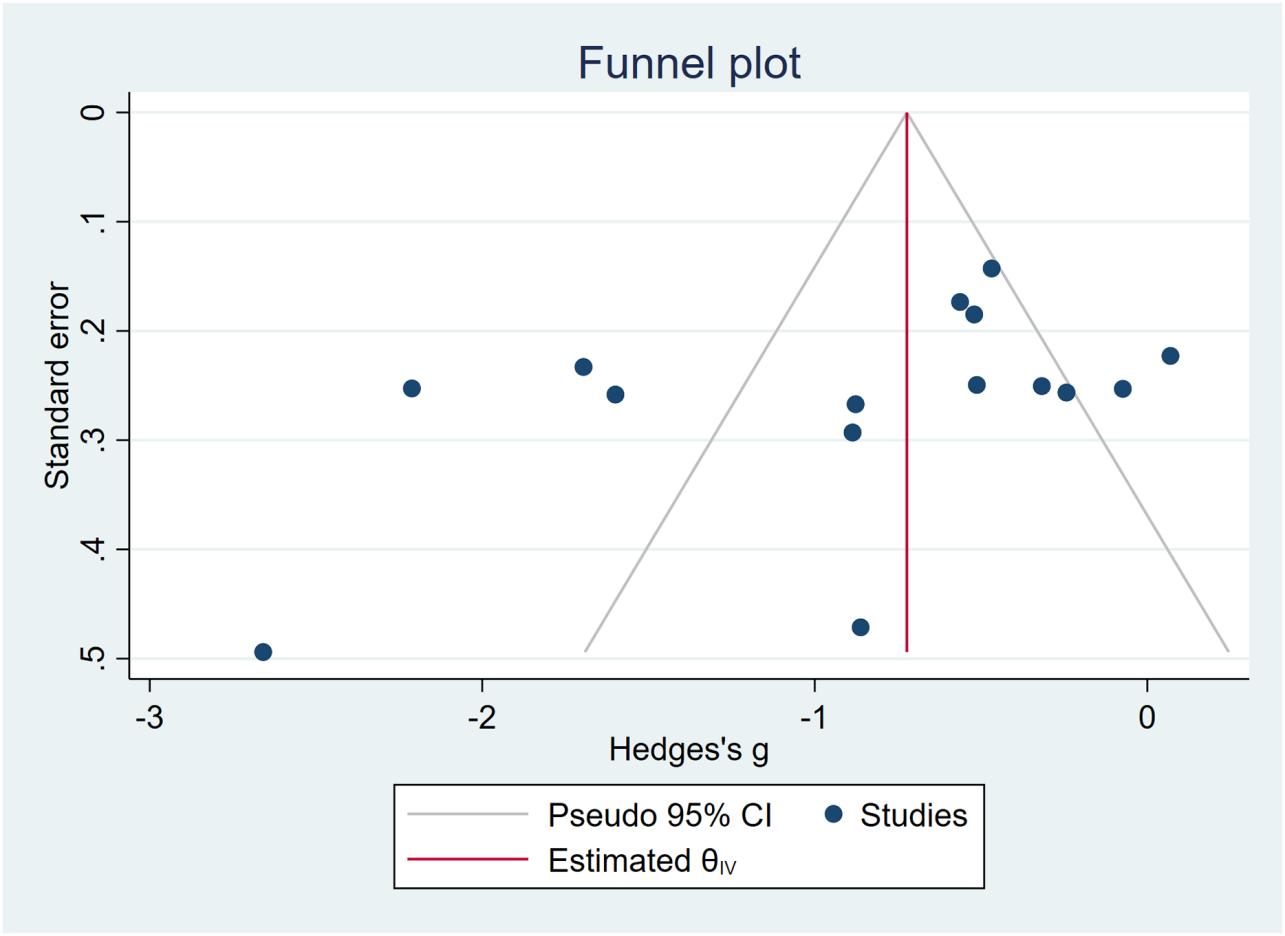
Funnel plots—depressive symptoms.

In the examination of the effects of mind-body exercises on depressive symptoms, Egger’s test yielded *t* = −1.91, *P* > 0.05, indicating the absence of significant publication bias, but small-study effects cannot be ruled out. The funnel plot exhibited no asymmetry, and trim-and-fill analysis confirmed no meaningful changes in the effect size or CI (Hedges’s *g* = −0.860, 95% CI [−1.241 to −0.478]). For a detailed graphical representation, refer to Fig. 6.

**Figure 7:**
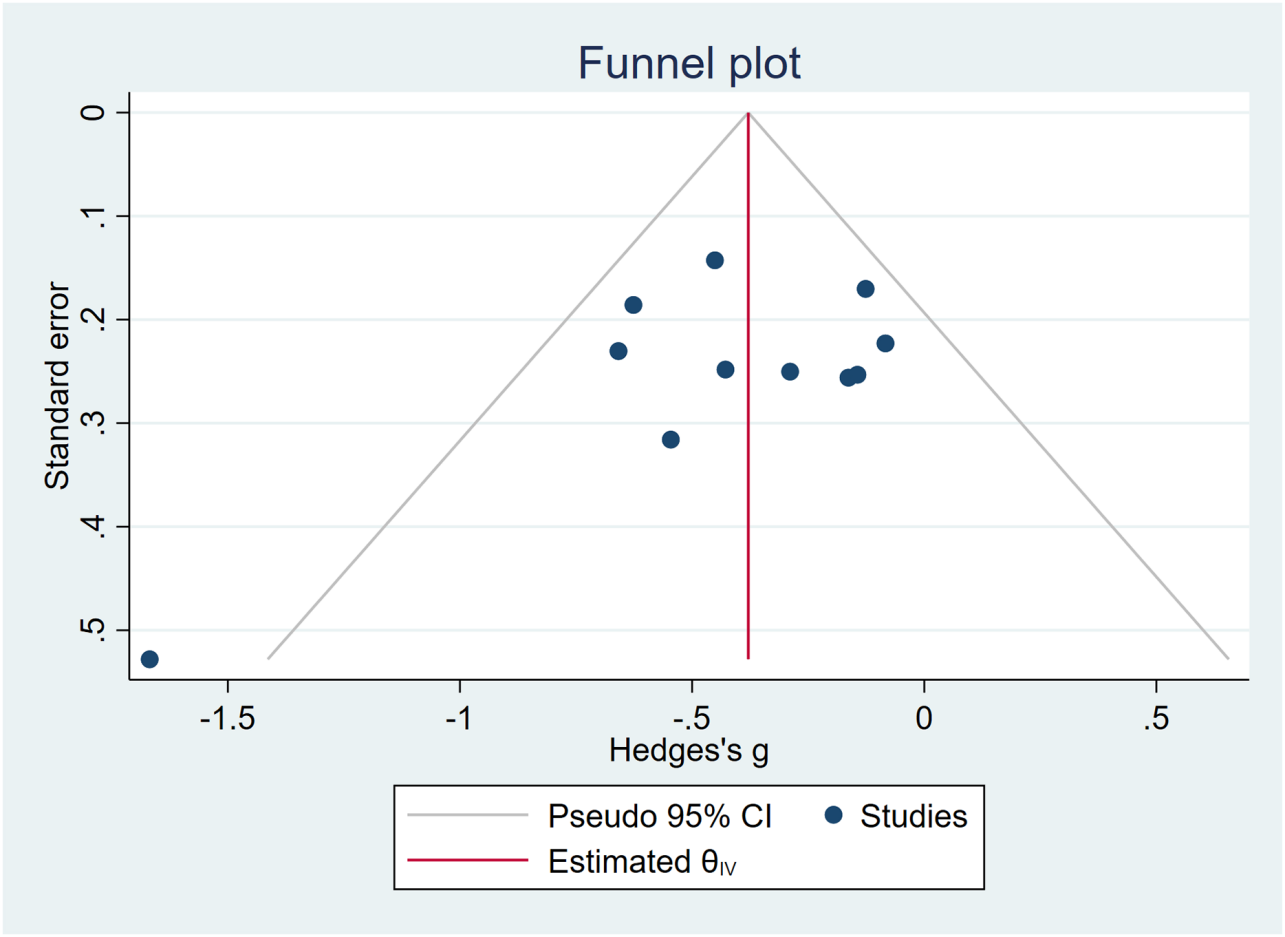
Funnel plots—anxiety symptoms.

In the assessment of the effects of mind-body exercises on anxiety symptoms, Egger’s test yielded *t* = −1.56, *P* > 0.05, indicating the absence of significant publication bias, but small-study effects cannot be ruled out. The funnel plot exhibited no asymmetry, and trim-and-fill analysis confirmed no notable changes in the effect size or CI (Hedges’s *g* = −0.381, 95% CI [−0.529 to −0.233]). For a comprehensive visualization, refer to Fig. 7.

### Meta-regression

To further explore the heterogeneity observed in the meta-analysis of depression outcomes (*I*^2^ = 87.98%), a meta-regression analysis was conducted using five continuous moderator variables: mean age, session duration (minutes), intervention period (weeks), frequency (times per week), and baseline depression severity. The results indicated that none of these variables significantly predicted the intervention effect (all *P*-values > 0.05). The overall model was not statistically significant (Wald *χ*^2^(5) = 5.21, *P* = 0.391), with a modest explanatory power for the observed heterogeneity (*R*^2^ = 5.90%). Residual heterogeneity remained substantial (*τ*^2^ = 0.4655, *I*^2^ = 87.98%). For further details, refer to Table 5.

**Table 5:** Meta-regression.

_meta_es	Coefficient	Std. err.	*z*	*P* > |z|	[95% CI]	
Age	−0.003561	0.0150399	0.24	0.813	−0.0259167	0.0330388
Session duration (min)	−0.0129022	0.0109759	−1.18	0.240	−0.0344146	0.0086102
Exercise period (week)	−0.0135999	0.0573009	−0.24	0.812	−0.1259076	0.0987079
Frequency (times/week)	−0.1029226	0.1022495	−1.01	0.314	−0.303328	0.0974828
Baseline depression severity	−0.3341733	0.2320215	−1.44	0.150	−0.7889272	0.2306906
_cons	0.8169919	1.21158	0.67	0.500	−1.557662	3.191646

**Note:**

Random-effects meta-regression. Number of obs = 15. Method: REML. Residual heterogeneity: tau^2^ = 0.4655; *I*^2^ (%) = 87.98; *H*^2^ = 8.32; *R*-squared (%) = 5.90; Wald chi^2^(5) = 5.21; Prob > chi2 = 0.3909.

### Evidence quality assessment

**Figure 8:**
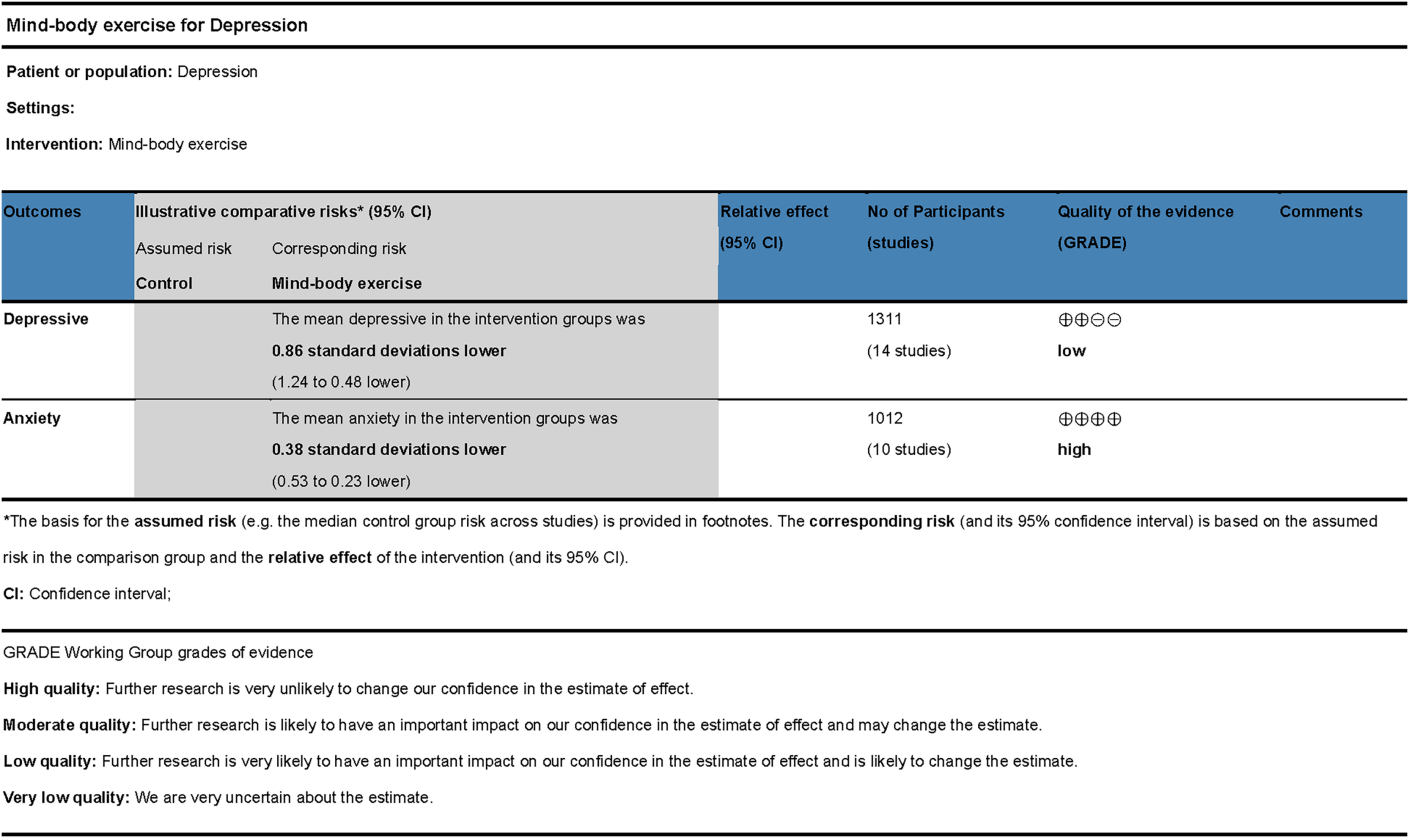
GRADE evidence quality assessment.

The GRADE approach was used to evaluate the quality of evidence regarding the effects of mind-body exercises on depressive and anxiety symptoms (refer to Fig. 8). Certain limitations were identified, primarily due to the absence of allocation concealment or blinding in some studies. Although sensitivity analysis did not detect significant sources of heterogeneity, potential inconsistencies cannot be ruled out. Consequently, the quality of evidence for studies on depression was rated as low, whereas the quality of evidence for studies on anxiety was rated as high.

## Discussion

This study employed a meta-analysis approach by synthesizing data from 15 included studies in order to evaluate the effects of mind-body exercises on depressive and anxiety symptoms. The results demonstrated that mind-body exercises significantly alleviated depressive and anxiety symptoms, aligning with prior research findings (Li et al., 2023a). Specifically, qigong has been identified as an effective intervention for major depressive disorder (Guo, Kong & Zhang, 2019), yielding notable psychological benefits and significantly mitigating depressive and anxiety symptoms (Zou et al., 2018b). Additionally, Brinsley et al. (2021) reported that yoga has a positive impact on depressive symptoms in individuals with mental disorders.

The overall quality of the included studies in this research was deemed good; however, some limitations persisted. Specifically, most studies lacked comprehensive reporting on the implementation of blinding or allocation concealment, which potentially influenced post-test outcomes. Egger’s test for the effects of mind-body exercises on depressive and anxiety symptoms indicated no significant publication bias. Additionally, the evidence quality assessment did not identify any notable reasons for downgrading due to indirectness or imprecision. The meta-analysis revealed that in studies evaluating the effects of mind-body exercises on depressive symptoms, *I*^2^ > 50% signified high heterogeneity among the included studies. Subgroup analyses suggested that factors such as exercise type, session duration, and exercise cycle might contribute to this heterogeneity. Sensitivity analysis demonstrated that sequential exclusion of individual studies did not substantially alter the effect size. Meta-regression showed that session duration, intervention period, frequency, and baseline depression severity were all non-significant predictors, and the underlying source of heterogeneity remained unexplained. Several factors may have contributed to the excessive heterogeneity. First, mind-body exercises inherently incorporate cultural elements, which may result in differential effectiveness across cultural contexts. Second, practices such as tai chi, qigong, and yoga exist in diverse forms, making it difficult to achieve standardized implementation. Finally, heterogeneity may also stem from unmeasured confounding variables, such as lifestyle quality, duration of symptoms, and individual psychological traits. This unexplained heterogeneity was deemed a factor for downgrading the evidence quality. Consequently, the quality of evidence for the effect of mind-body exercises on depression was rated as low, whereas the quality of evidence for its effect on anxiety was rated as high.

Previous studies have indicated that mind-body exercises may promote positive changes by enhancing physiological proprioception. These practices integrate movement and breathing control with specific states of consciousness (Zou et al., 2018a), and the meditative state induced by heightened self-awareness and proprioception is hypothesized to involve key brain regions, including the insula, medial prefrontal cortex, posterior cingulate cortex, and precuneus (Yeung, Campbell & Chan, 2019), thereby contributing to improvements in depressive and anxiety symptoms.

In studies on depression, exercise type, single-session duration, frequency, and baseline depression severity were identified as significant moderators, which is largely consistent with previous findings (Brinsley et al., 2021; Wang et al., 2022). In the meta-analysis by Wang et al. (2022), exercise frequency, session duration, and exercise cycle were identified as potential moderators influencing depression among adolescents. However, unlike the present study, which focused exclusively on mind-body exercise, Wang et al. (2022) analyzed general physical activity, and the population characteristics also differed. Similarly, Brinsley et al. (2021) reported that exercise frequency may be a moderator for depressive symptoms, although their study concentrated on individuals with psychiatric disorders, which differs from the population examined here. In terms of effect sizes, a large and most pronounced effect was observed for sessions lasting 31–60 min (Hedges’s *g* = –1.315, 95% CI [–1.874 to –0.755]). For exercise frequency, significant effects were found for both three times per week and ≥ four times per week, with the strongest effect observed at three times per week (Hedges’s *g* = –2.045, 95% CI [–3.070 to –1.020]). Regarding exercise type, qigong demonstrated the strongest effect (Hedges’s *g* = –1.228, 95% CI [–1.805 to –0.650]). In terms of baseline depression severity, mind-body exercise showed large effects for both highly and mildly depressed individuals, with the greatest effect among those with high baseline depression (Hedges’s *g* = –1.176, 95% CI [–2.157 to –0.195]). A large effect was also found for interventions lasting 9–12 weeks (Hedges’s *g* = –1.104, 95% CI [–2.044 to –0.164]), although intervention cycle was not identified as a moderator. These findings suggest that exercise type, session duration, frequency, and baseline depression severity significantly influenced the effects. Among the various types of mind-body exercises, qigong demonstrated the most pronounced effects, aligning with the findings of Gill et al. (2020). This may be attributed to qigong’s ability to alleviate depression by modulating the autonomic nervous system, particularly by enhancing parasympathetic activity (So et al., 2019). From the perspective of session duration, the optimal intervention effect was observed within 31–60 min, with no significant improvement noted for sessions exceeding 60 min. This finding suggests that increasing practice time within this range effectively enhances outcomes, with the 31–60 min window yielding the most favorable results. Sessions exceeding 60 min failed to yield additional benefits, consistent with the conclusions of Johansson & Hassmén (2008), who found that 30 min was sufficient in inducing positive psychological responses. While extended qigong practice may offer physiological advantages (Ai, 2003), excessively prolonged sessions are more difficult to sustain and may induce fatigue, ultimately diminishing the effectiveness of the intervention. Regarding exercise cycles, the optimal effect was achieved within a 9–12 week cycle, with a decline in effectiveness observed beyond 12 weeks. This suggests that the 9–12 week duration is ideal, and extending the cycle further does not produce additional benefits (Li et al., 2023b). In terms of frequency, practicing three times per week yielded the most favorable outcomes, while higher frequencies led to diminished effects. This decline could be due to fatigue associated with more frequent practice, which may limit further improvements in outcomes. From the perspective of baseline depression severity, mind-body exercise was effective for individuals with high, low, and normal levels of depressive symptoms, with the greatest improvement observed in those with severe depression. This may be because participants with higher baseline symptom scores have more room for improvement, resulting in larger observed effect sizes.

In studies on anxiety, neither exercise-related variables nor baseline anxiety severity were identified as significant moderators, which is consistent with previous findings (Yin et al., 2023). In the meta-analysis conducted by Yin et al. (2023), no significant moderating factors were identified; however, it should be noted that their study focused on tai chi and non-mindfulness-based exercises, whereas the present study centers on mind-body exercises. A moderate and the most pronounced effect was observed for single-session durations of 31–60 min (Hedges’s *g* = –0.507, 95% CI [–0.780 to –0.234]). Practicing at a frequency of ≥ four times per week also yielded a moderate and most pronounced effect (Hedges’s *g* = –0.450, 95% CI [–0.672 to –0.229]). Regarding exercise type, both qigong and tai chi produced moderate effects, with tai chi showing the most notable effect size (Hedges’s *g* = –0.593, 95% CI [–1.407 to 0.221]). In the subgroup analysis based on baseline anxiety levels, mind-body exercise had a moderate effect in participants with normal anxiety levels, and this subgroup showed the strongest effect size (Hedges’s *g* = –0.639, 95% CI [–0.923 to –0.356]). A large and most pronounced effect size was observed when the exercise cycle was ≤8 weeks (Hedges’s *g* = –0.961, 95% CI [–2.164 to 0.243]). This outcome may be attributed to longer exercise cycles providing individuals with more opportunities to build supportive relationships with peers or exercise professionals (Zhang et al., 2023b), which may further mitigate anxiety symptoms. In terms of exercise frequency, practicing at least four times per week exhibited a dose-response relationship with intervention effectiveness. A plausible explanation is that the relaxation effects of qigong can enhance autonomic nervous system function and regulate the vagus nerve (Sun et al., 2024), thereby alleviating anxiety symptoms. The subgroup analysis in this study revealed that the effects of mind-body exercises on alleviating depression and anxiety were influenced by exercise type, session duration, frequency, and exercise cycle, with varying impacts on the two conditions. This study’s findings differ from those of previous research (Martínez-Calderon et al., 2023). For instance, Wang et al. (2013) concluded that certain mind-body exercises showed no significant effects on anxiety, and Gill et al. (2020) suggested limited or insignificant effects on depressive symptoms. In terms of baseline anxiety severity, mind-body exercise produced small-to-moderate effects for individuals with high and moderate anxiety, and a moderate effect for those with normal anxiety levels. These differences may be attributed to variability in exercise type, duration, frequency, intervention cycle, and baseline anxiety severity across studies.

This study has several limitations related to the characteristics of the included literature. First, all outcome measures were based on self-reported questionnaires, which may be influenced by subjective bias. Second, there were inconsistencies in the standardization of interventions and selection of assessment tools across studies, potentially affecting the comparability and accuracy of the results. Third, mind-body exercises emphasize the integration of physical and mental engagement, but it is difficult for researchers to verify whether participants truly practiced with mindful awareness, making it challenging to assess cognitive engagement. The included studies also spanned a wide time range, and earlier studies may be limited by outdated designs and methodological constraints. Although the overall sample size was relatively large, some subgroup analyses were based on a limited number of studies and participants, reducing their clinical interpretability. This study did not perform detailed analyses based on region, culture, or ethnicity, which may have affected the generalizability of the findings. Although all included studies used standardized scales to assess the severity of depression and anxiety, most failed to specify participant diagnostic status, such as whether symptoms were from clinically diagnosed major depressive disorder or whether participants presented with comorbid depression and anxiety. This may have contributed to the high heterogeneity observed in the meta-analysis on depression. Studies comparing mind-body exercise with active control groups were excluded in order to better isolate the specific effects of the intervention. However, this exclusion may compromise the external validity of the findings and introduces a potential risk of bias, as comparisons against passive controls (*e.g*., waitlist or no-treatment groups) often produce inflated effect sizes due to the absence of placebo or engagement effects. Consequently, the observed benefits may not accurately reflect the relative advantage of mind-body exercise over other active interventions. Future meta-analyses should consider including both active and passive control groups and perform subgroup analyses to more precisely assess the comparative efficacy of mind-body exercises relative to other exercise modalities. Finally, most of the studies included in this meta-analysis did not conduct follow-up assessments to evaluate the long-term effects of the interventions, making it difficult to determine the sustained impact of mind-body exercise on mental health.

## Conclusion

Mind-body exercises demonstrate significant effectiveness in alleviating both depressive and anxiety symptoms. The improvements observed were influenced by multiple exercise-related factors, including exercise type, session duration, frequency, exercise cycle, and baseline symptom severity. Subgroup analyses revealed that individuals with higher baseline levels of depressive or anxiety symptoms tend to experience greater benefits from mind-body exercises, suggesting that baseline severity is an important moderator of intervention effects. Current evidence suggests that for individuals with depressive symptoms, optimal benefits are achieved by practicing qigong for 31–60 min per session, three times per week, over a cycle of 9–12 weeks, with the most pronounced improvements observed among those with high baseline depression severity. For individuals experiencing anxiety symptoms, qigong practice for 31–60 min per session, at least four times per week, for a duration of 16 weeks or longer, was associated with the most favorable outcomes, particularly among those with higher baseline anxiety severity. These findings highlight the necessity of tailoring mind-body exercise programs to individual baseline symptom profiles and optimizing key exercise parameters to maximize mental health benefits.

## Supplemental Information

10.7717/peerj.20570/supp-1 Supplemental Information 1 PRISMA checklist.

10.7717/peerj.20570/supp-2 Supplemental Information 2 Meta-analysis data.

## Additional Information and Declarations

### Data Availability

The following information was supplied regarding data availability:

The raw data is available in the Supplemental File.
