# Supplementary material for: A systematic review and meta-analysis of the effects of mind-body exercise on depressed and anxious individuals
Source: PeerJ. 2026 Jan 6;14:e20570. doi: 10.7717/peerj.20570 (PMC12786122; doi:10.7717/peerj.20570)
Supplement: Supplemental Information 2 [file peerj-14-20570-s002.docx]

***Depression***

| ***Study*** | ***Sample size(T)*** | ***Mean*** | ***SD*** | ***Sample size(C)*** | ***Mean*** | ***SD*** |
| --- | --- | --- | --- | --- | --- | --- |
| Suhui Ma, 2010 | 50 | -8.98 | 4.2568650 | 50 | 0.22 | 3.9954099 |
| Suhui Ma, 2011 | 49 | -6.88 | 3.8801160 | 50 | -0.04 | 4.1201456 |
| Yvonne W. Y, 2012 | 34 | -3.35 | 4.7283507 | 31 | -0.52 | 6.1562732 |
| Tiffany Field, 2013 | 40 | -9.20 | 9.7811042 | 39 | -9.90 | 10.1133580 |
| JessieS.M.Chan, 2013 | 72 | -1.40 | 2.8000000 | 65 | 0.40 | 3.5538711 |
| Melissa M. Buttner, 2015 | 23 | -11.46 | 5.6229796 | 27 | -6.82 | 4.7199258 |
| Xiang Chen, 2016 | 15 | -7.9 | 2.1283797 | 15 | -2.3 | 1.9672316 |
| Zhenlei Ma, 2016 | 38 | -1.97 | 1.3352528 | 40 | -0.03 | 1.0578752 |
| Liang Gao, 2016 | 149 | -2.85 | 6.1752976 | 37 | 0.51 | 7.3873405 |
| Qiang Liu, 2016 | 32 | -0.16 | 0.5350701 | 31 | 0.02 | 0.5855766 |
| Guangjian Fu, 2016 | 100 | -3.30 | 9.8905460 | 100 | 1.68 | 11.2655630 |
| Bin Zhao, 2017 | 30 | -18.43 | 3.2729345 | 30 | -15.46 | 3.4098240 |
| Zhengchun Hua, 2021 | 60 | -2.77 | 4.8101247 | 20 | -1.50 | 6.1681764 |
| Zhengchun Hua(1), 2021 | 66 | -1.88 | 4.7763270 | 20 | -1.50 | 6.1681764 |
| Jianwei Zhang, 2023 | 9 | -15.34 | 9.0459881 | 9 | -7.06 | 9.2490216 |

***Anxiety***

| ***Study*** | ***Sample size(T)*** | ***Mean*** | ***SD*** | ***Sample size(C)*** | ***Mean*** | ***SD*** |
| --- | --- | --- | --- | --- | --- | --- |
| Yvonne W. Y, 2012 | 34 | -3.53 | 5.6682361 | 31 | -0.84 | 6.7531992 |
| Tiffany Field, 2013 | 40 | -6.7 | 8.4717177 | 39 | -6 | 8.1166496 |
| JessieS.M.Chan, 2013 | 72 | -2.3 | 2.8160256 | 65 | -1.9 | 3.4871192 |
| Liang Gao, 2016 | 149 | -3.81 | 6.4950058 | 37 | 0.35 | 7.0835796 |
| Qiang Liu, 2016 | 32 | -0.18 | 0.3477068 | 31 | -0.07 | 0.4033609 |
| Guangjian Fu, 2016 | 100 | -3.26 | 9.9005454 | 100 | 1.43 | 10.807789 |
| Shirley Telles, 2016 | 20 | -11.25 | 11.190661 | 20 | -5 | 11.257282 |
| Zhenlei Ma, 2016 | 38 | -5.12 | 6.318829 | 40 | -0.31 | 8.0016998 |
| Zhengchun Hua, 2021 | 60 | -2.53 | 5.585329 | 20 | -1.6 | 5.8178432 |
| Zhengchun Hua(1), 2021 | 66 | -2.42 | 5.5958109 | 20 | -1.6 | 5.8178432 |
| Jianwei Zhang, 2023 | 9 | -25.9 | 11.297225 | 9 | -5.7 | 11.762517 |
